# Toward an integrated ethical review process: an animal-centered research framework for the refinement of research procedures

**DOI:** 10.3389/fvets.2024.1343735

**Published:** 2024-04-17

**Authors:** Eleonora Nannoni, Clara Mancini

**Affiliations:** ^1^Department of Veterinary Medical Sciences, University of Bologna, Ozzano Emilia (BO), Italy; ^2^Animal-Computer Interaction Lab, School of Computing and Communications, The Open University, Milton Keynes, United Kingdom

**Keywords:** animal research, animal-centered research ethics, refinement, ethical review, relevance, impartiality, welfare, consent

## Abstract

The involvement of animals in research procedures that can harm them and to which they are deemed unable to consent raises fundamental ethical dilemmas. While current ethical review processes emphasize the application of the 3Rs (Replacement, Reduction, and Refinement), grounded in a human-centered utilitarian ethical approach, a comprehensive ethical review also involves a harm-benefit analysis and the consideration of wider ethical issues. Nevertheless, to our knowledge, approaches are still needed to facilitate the integrative assessment and iterative revision of research designs to improve their ethical value or to identify cases in which using animals is irremediably unethical. Additionally, frameworks are lacking that explicitly include an animal-centered perspective into the ethical review process beyond welfare concerns, failing to cover broader ethical considerations (such as consent). In previous work we proposed an Animal-Centered Research framework (ACRf) comprising four *animal-centered research* principles (*relevance*, *impartiality*, *welfare* and *consent*) which could help researchers and ethical review bodies apprise research designs from an animal-centered perspective. This paper builds on and further develops our previous work by contextualizing the ACRf within the bigger picture of animal research ethical review and by illustrating how the ACRf could be operationalized within current ethical review processes. We contribute an extended framework that integrates the application of the ACRf principles within the ethical review process. To this end, we present findings from a theoretical case study focusing on the ethical review of a research protocol on the study of stress response in pigs. We discuss how our extended framework could be easily applied to facilitate a holistic approach to the ethical review process, and inform an iterative process of refinement, to support the development of research designs that are both more ethical and scientifically valid.

## 1 Introduction

The involvement of animals in research procedures that can harm them and to which they are deemed unable to consent raises fundamental ethical dilemmas. The framework of the 3Rs (Replacement, Reduction and Refinement), proposed over 60 years ago (1), addresses these dilemmas, and its principles are widely regarded as the gold standard for humane animal research, and the best compromise between animal welfare and research aims. However, the 3Rs framework is based on the assumption that animals are essentially instruments in a research apparatus (albeit needing protection), and is grounded in a human-centered utilitarian ethical approach, implying that the use of animals in research is legitimate when it is intended to achieve a greater good for society (2). This is in contrast with frameworks regulating the involvement of humans in research, which protect participants’ autonomy and wellbeing, and require their just treatment (3). Additionally, within the framework of the 3Rs, consent to carry out a procedure is usually not given by the animals involved, but provided by human actors. The most common actors consenting to the use of animals are institutional ethical review bodies who authorize (or forbid) research protocols, although in some cases owners are also required to provide consent for their animals (e.g., when pets are involved). Usually, these actors are deemed capable of understanding the wider implications of animals’ involvement in experimental procedures and have the legal authority to decide whether their involvement is warranted.

Mancini and Nannoni (4) point out how, despite sensibly contributing to reducing the number of unnecessary procedures carried out on animals and achieving an overall improvement in research practices, over the years various limitations of the 3Rs framework have emerged. For instance, “Replacement” states that, whenever possible, animal use in research should be replaced with alternative methods, or more complex species should be replaced with species deemed less sentient. This approach is based on the assumption that animals are substitutable components of an experimental set-up and that being involved in research is always detrimental to them, without acknowledging that in some cases it might be beneficial for individual animals to be involved as stakeholders able of expressing their (and their species’) interests in research outcomes (5). Moreover, replacement of more complex species with less complex ones may result in the use of more individual animals (6), thus contravening the principle of “Reduction”. This requires researchers to limit the number of animals involved in trials to the minimum necessary for statistical significance, although reducing numbers may mean that fewer animals are subjected to more severe or multiple procedures (causing greater harm to the individuals involved), in turn contravening the principle of “Refinement”. This requires that research procedures be designed and adapted to minimize any negative impact (pain, suffering or distress) on the animals involved, benefitting both animals and science. Refinement might include a very broad set of strategies, with differing effectiveness for improving animal welfare (or for limiting negative impacts on welfare). However, to our knowledge, no standardized approaches on how to plan for or report on refinements are available to support researchers and ethical review bodies in this regard. Where available, refinement guidelines [e.g., (7, 8)] only focus on welfare harms, with no consideration for broader *ethical harms* (e.g., subjecting animals to any procedures without their consent, regardless of whether these may have any welfare impacts).

To address these limitations, Mancini and Nannoni (4) proposed an Animal-Centered Research framework (ACRf) comprising four *animal-centered research* principles: *relevance* (of the research to the individuals involved in procedures, or at least to the species—provided that the individual suffers no harm), *impartiality* (of treatment regardless of categorizations such as species), *welfare* (including physical and psychological wellbeing) and *consent* (to be provided both by the individual animals involved and their guardians). Through illustrative examples based on published research, the authors discuss how their proposed principles could help assess the extent to which research protocols might conform with an animal-centered ethics, helping to identify cases in which refinements or alternative methods might be needed.

These principles were derived from the field of Animal-Computer Interaction (5, 9), whose aims include the study and design of animal-centered technology and animal-centered research methods to enhance animals’ welfare, increase their autonomy, support their activities and improve their standing in human society. The field’s animal-centered perspective is grounded in both ethical and scientific considerations, whereby technologies designed to be consistent with animals’ characteristics, usability and experience requirements are more likely to be effective [e.g., biotelemetry devices designed to optimize wearability produce lower welfare impact and provide greater data reliability—Paci et al. (9); computing interfaces with which animals are expected to interact to complete given tasks need to be consistent with their sensory, cognitive, physical and behavioral characteristics—Ruge et al. (10)]. Similarly, Mancini and Nannoni (4) argued that research designs which take an animal-centered perspective are more likely to deliver scientifically valid results and, thus, greater societal benefits. However, the authors did not address the issue of how their animal-centered framework could be operationalized and integrated within the ethical review process that researchers are required to undertake prior to conducting any research involving animals.

The work presented here builds on and further develops the previous work of Mancini and Nannoni (4) by contextualizing the ACRf within the bigger picture of animal research ethical review and by illustrating how their ACRf could be operationalized within current ethical review processes. This paper contributes an extended framework that integrates the application of the ACRf principles within the ethical review process to facilitate a more holistic approach, and inform an iterative process of refinement, to support the development of research designs that are both more ethical and scientifically valid. To this end, the remainder of the paper discusses different aspects of the ethical review process and related limitations. The paper then summarizes the ACRf and its four constituting principles, including the scoring system previously proposed by Mancini and Nannoni (4) to assess research procedures from an animal-centered perspective. To demonstrate the application of the framework, its integration within the ethical review process, and how this could inform refinements to research plans, the paper presents findings from a theoretical case study focusing on the ethical review of a research protocol on the study of stress response in pigs. We discuss how, by facilitating a more integrative approach to the ethical review process and informing an iterative process of refinement, the framework could provide a simple approach to assessing the ethical value of research procedures from an animal-centered perspective and lead to the development of research designs that are both more ethical and scientifically valid. We conclude by outlining plans for future work, involving the empirical validation of our proposed approach by researchers and ethical review bodies when preparing and assessing a range of research proposals.

## 2 Background

### 2.1 The ethical review of animal research

As highlighted by the UK’s Royal Society for the Prevention of Cruelty to Animals (RSPCA), a comprehensive ethical review is not limited to the application of the 3Rs, but involves consideration of the following aspects (11):

- *Harm-benefit analysis*—this involves considering the potential benefits of a research project against the expected harms to animals, and is at the basis of ethical frameworks underpinning regulations on animal experiments in the European Union and part of the decision-making process by individual researchers and other bodies involved (e.g., competent authorities, ethics or animal care and use committees).
- *Three Rs implementation*—this includes Replacement (as the main objective), Reduction (optimizing numbers and avoiding wasting animals by ensuring effective experimental design and statistical analysis), and Refinement for immediate benefit to a large number of animals; such benefit pertains to three main areas: (1) refining housing, husbandry and procedures to reduce lifetime harms to laboratory animals; (2) promoting effective welfare assessment of laboratory animals, including pain, suffering and distress, as well as positive welfare; (3) tackling severe suffering with the support of regulators and the scientific community.
- *Wider ethical issues*—this entails determining what, all things considered, should be done following wider ethical discussions, not only among individual research groups on issues directly relating to specific project licenses, but also among the wider scientific community on broader issues relating to the appropriateness of animal research.

Along similar lines, DeGrazia and Beauchamp (12) encourage researchers and other stakeholders to look “beyond the 3Rs” when developing research plans. The authors propose that the 3Rs framework should be implemented by balancing 3 principles of social benefit (i.e., that no alternative method to the use of animals exists, that the expected net benefit of the research to society is significant, and that the value of the research is sufficient to justify any harm to the animals involved) against 3 principles of animal welfare (i.e., that no unnecessary harm should be caused, that the basic needs of the animals involved should be respected, that upper limits to what is admissible harm should be set).

In other words, an ethical approach to the involvement of animals in research requires consideration of a wide range of issues, respectively concerning the design of procedures and their welfare impacts, the balance between harms to animals and benefits to society, and the very ethical legitimacy, or otherwise, of using animals for research purposes. These aspects are clearly interlinked. For example, the legitimacy of using animals in research is open to question precisely because procedures may harm them without their consent and because the individuals being used usually suffer the harms without enjoying the benefits. Nevertheless, to our knowledge, frameworks are still lacking to explicitly integrate the different aspects of the ethical review process and to facilitate the integrative assessment and iterative revision of research designs to improve their ethical value (or to identify cases in which using animals is irremediably unethical, and the development and deployment of alternative methods should be prioritized). Importantly, to our knowledge, frameworks are also lacking to integrate the animals’ perspective in the ethical review process beyond welfare concerns to include broader ethical considerations (such as consent). An approach that reflected the interconnection among the different aspects of the ethical review process and that accounted for the animals’ perspective in relation to those aspects, we suggest, would help researchers and review bodies take the holistic approach that has been advocated to addressing the complexities surrounding one of the most controversial aspects of human-animal relationships.

### 2.2 The ethical review process and some ethical limitations

Debates on the legitimacy of animal research leverage both moral and scientific arguments. On the one hand, those against may argue that using animals for human purposes in procedures that harm them and to which they have not consented is not morally acceptable; or that the use of animals as models for biological processes they are expected to substitute for does not yield reproducible results and thus is not scientifically reliable. On the other hand, those in favor may argue that the harm inflicted upon animals in procedures is necessary to achieve important societal benefits, including reducing human and other animals’ suffering; or that in many cases the use of animals is the only way to obtain scientifically reliable data. These debates take place within dynamic socio-cultural contexts and, as societal sensibilities evolve, the moral and scientific values reflected in the policies that regulate the use of animals in research also change. Overall, mounting evidence on the complexity of animals’ experience has been strengthening the moral argument against animal research, leading to stronger protections for some species (e.g., cephalopods) or to the prohibition of using animals for some tests (e.g., cosmetics). At the same time, the increasing availability of alternative methods has been weakening the scientific argument in favor of animal experimentation in some cases, leading to the recommendation that such alternative methods should be used wherever possible. Nevertheless, in many cases, the use of animals in research is still deemed necessary by some scientists and by regulators, for fundamental (e.g., physiology), translational (e.g., use of animal models of disease) and applied (e.g., vaccine development) research, for some educational purposes (e.g., surgery training), and for meeting regulatory requirements (e.g., toxicology tests).

However, as highlighted by UK’s Animal Procedure Committee (7), despite philosophical divergences on the relation between humans and other animals, there is consensus that the use of animals in scientific procedures is a matter of moral significance and that such use is only morally defensible under specific circumstances provided strong scientific grounds. In particular, according to the APC, “*procedures that inflict injury on animals for reasons other than for their own good require robust defense*” with regards to “*the rights and wrongs of the actions involved; the worth, or otherwise, of the motivations; and the actual and/or potential consequences*” (p. 9). In other words, the nature and extent of procedures’ impact on the animals involved (e.g., mild temporary discomfort vs. severe prolonged suffering) need to be carefully weighed against the potential benefits of the research. However, demonstrating that the benefits of using animals in research outweigh the harms is notoriously challenging, as acknowledged by policy advisory working groups such as the American Association for Laboratory Animal Science-Federation of European Laboratory Animal Science Association (AALAS-FELASA) (13). This is because in much animal research (e.g., medical research, regulatory tests), the harms inflicted upon animals are both certain and immediate, while the benefits to society are uncertain and may only come to fruition in the long term (especially in the case of fundamental research).

There is also consensus that determination of the harm-benefit balance is ultimately a judgment call based largely on probability and qualitative analysis of a wide range of variables (14). In this regard, when assessing potential harms, researchers and ethical review bodies are asked to consider *project related harms* (those specific to the procedures), *contingent harms* (including husbandry, care practices, transportation) and *cumulative effects* (resulting from the accumulation of all the effects produced by both procedures and husbandry) to assess how a procedure may affect animals in practice (8). Researchers are also expected to assess the actual harms of a procedure once this has been completed and when the full extent of its impact has become apparent. Procedures are classified according to their severity: *non-recovery* (if undertaken entirely under anesthetic from which the animal is not allowed to recover), *mild* (if producing a modest, temporary impact), *moderate* (if producing a modest prolonged impact or a more significant but temporary impact), *severe* (if producing significant and lasting impact).

To help researchers and ethical review bodies discriminate severity levels based on the consistent application of meaningful criteria, various frameworks have been proposed over the years [e.g., (15, 16)]. One such framework was proposed by Mellor and Reid (17), and later revised by Mellor (18), who adapted the principles of the Five Freedoms previously developed by the Farm Animal Welfare Council (19) and widely used in a variety of contexts in which managed animals are kept. The principles state that, for good welfare to be present, animals should be *free from hunger or thirst* (having ready access to fresh water and a diet to maintain full health and vigor), *from discomfort* (having an appropriate environment including shelter and a comfortable resting area), *from pain, injury or disease* (through prevention or rapid diagnosis and treatment); they should be *free to express (most) normal behavior* (having sufficient space, proper facilities and the company of the animal’s own kind), and *from fear and distress* (being provided with conditions and treatment which avoid mental suffering). These principles have been extensively used in the assessment of potential harms of research procedures and have also informed practical tools to facilitate the weighing of harms against benefits [e.g., (13)], providing benchmarks against which to refine procedures to reduce related harms.

Notwithstanding their established usefulness, these tools reflect some key assumptions implicit in the way in which the harm-benefit analysis of proposed animal research is generally approached. Firstly, there seems to be an assumption that harms to the animals only pertain to welfare variables (e.g., to what extent an animal might suffer hunger, discomfort, restriction, fear). Of course, the extent to which a procedure is expected to impact on an animal’s welfare is a key ethical consideration. However, there is no explicit criterion to reflect the possibility that the extent to which an animal might or might not be allowed to consent or dissent to a procedure might be part of the harm-benefit equation. The issue is not simply whether an animal might be physically forced or trained to voluntarily undergo a procedure (although the latter option is certainly more consensual), but more fundamentally the extent to which they are enabled to exert their agency effectively to advance their own best interests above and beyond the immediate effect on welfare variables (although it is widely acknowledged that the ability to exert agency contributes to good welfare) (20, 21).

Secondly, there seems to be an assumption that being involved in research can never have benefits for the individual animals in question, which places animals’ interests on one side and societal interests on the other side of the harm-benefit equation, pitting one against the other by default. However, it may be more accurate to explicitly acknowledge that the positioning of animals’ interests across the harm-benefit divide depends on whether the research is relevant to the individuals involved. It could be argued that taking part in research that directly benefits them significantly limits the potential harms to the animals involved. In this regard, at least in principle, if an initially harmful research design could be refined to a point at which the research became beneficial for the animals involved, animals’ and society’s interests could find themselves onto the same side of the harm-benefit equation, which could significantly increase the ethical acceptability of the research.

Thirdly, there is a systemic assumption that treatment standards (in terms of both permissible experimental practices and acceptable husbandry practices) for animal research subjects do not need to be as high as they need to be for human research subjects. Indeed, if they had to be, animals would, for example, need to be allowed to withdraw from any procedure at any time and some procedures could simply not be carried out to begin with. However, although certain procedures may be regarded as necessary to achieve the desired benefits, we argue that their ethical review should assess how the treatment standards envisaged for animal research subjects might compare to those that would normally be afforded to human research subjects. Regardless of whether such standards could in practice be afforded to animals, they would provide a benchmark against which the ethics of a procedure could be assessed and related refinements could be measured.

In other words, while animal welfare considerations are of critical importance for the ethical review of proposed animal research and the related harm-benefit assessment, we argue that other aspects should also be considered, which are likely to affect the welfare of the animals involved but, from an ethical perspective, are important regardless. These might be regarded as *ethical harms or benefits* and include the issues of *consent*, *relevance* and *impartiality*, in addition to the issue of *welfare*, as envisaged by the ACRf (4). In this paper we demonstrate how an ethical review process integrating the application of the ACRf principles could facilitate a holistic ethical appraisal of proposed (or executed) animal research designs and could be leveraged to inform their refinement in order to support the development of research protocols and practices that treat animals as research participants rather than mere experimental subjects, and to promote research that is more animal-centered, on both ethical and scientific grounds.

### 2.3 From humane animal research to animal-centered research

Within some fields of applied research, such as Interaction Design (22), which focuses on the design of interactive systems, the involvement of (human) participants is deemed fundamental to establish the requirements that interactive products need to satisfy based on stakeholders’ characteristics, activities, environments and expectations. Stakeholders (particularly target users) are therefore central to the design and development process, which is structured as an iterative prototyping and evaluation cycle (23). Each iteration leads to a more comprehensive articulation of design requirements and to the refinement of the design solution whose aim is to meet those requirements. This perspective has recently been extended to the study and design of interactive systems targeted to nonhuman animals, within the field of Animal-Computer Interaction (ACI) (5). The ethical approach of ACI recognizes the centrality of animals’ capabilities for the design of interactive systems and the importance of animals’ dignified participation in research to ensure the effectiveness of said systems, thus regarding the animals involved as legitimate stakeholders within the research process (5). This approach has informed a range of applications in different domains, including, for example: the design of dog-friendly interfaces enabling mobility assistance dogs to operate domestic appliances on behalf of their assisted humans [e.g., (24)]; the design of wearable biotelemetry devices for wild animals to minimize the impact of the technology on animal wearers [e.g., (9)]; the design of digital enrichment devices for captive animals [e.g., (25)]; apparatuses for behavioral or cognitive research on animals [e.g., (26)]. ACI’s ethical approach has also informed the development of animal-centered design frameworks, such as the one proposed by Webber, Cobb, and Coe (27) to define animal-centric objectives and refine them through the course of a project, combining the “Five Domains of Animal Welfare” model (Nutrition, Environment, Physical Health, and Behavioral interactions, affecting together the final domain, Mental State) (28) and the “Coe Individual Competence” model (offering animals opportunities entailing Choice, Control, Variety, and Complexity, which all contribute to the development of Competence) (29). At least to some extent, these frameworks are relevant to any application context in which technologies targeted to animals are developed, including for example the field of precision livestock farming, where the importance of designing technology to improve animal welfare and productivity has been recognized [e.g., (30, 31)].

Generally speaking, ACI’s perspective departs from a utilitarian, anthropocentric approach to research, whose aim is humaneness, whereby procedures are carried out only after a harm-benefit analysis has confirmed the societal importance of the research and with care to minimizing animal suffering, but whereby the achievement of research results is nevertheless prioritized. Instead, as mentioned above, ACI takes an animal-centered approach, whose aim is enabling animals to participate in the design process as legitimate stakeholders and contributors, whereby relevance to partakers, impartial treatment of partakers, partakers’ welfare prioritization and partakers’ consent constitute important principles for animals’ involvement in research (5).^1^ Mancini and Nannoni’s (4) ACRf constitutes a development of these ACI research ethics principles for application to other kinds of animal research, with a view to fostering better research practices while prioritizing (individual) research participants’ autonomy and welfare.

### 2.4 Principle of the animal-centered research framework

The ACRf’s principles (4) can be summarized as follows:

- *Relevance:* This implies that animals should be involved in any research procedures only if said procedures are directly relevant and beneficial to them. Thus, when conducting a harm-benefit analysis, a separation between stakeholders, whereby those who suffer the negative consequences of a procedure are not those who stand to gain its expected benefit, should be avoided whenever possible. Of course, depending on the situation, relevance and its related benefits should be considered, not only in terms of the individual animal involved in a specific research trial but also, for example, in terms of the category or species to which the animal belongs (provided, as mentioned above, that the individuals involved do not suffer harms). One example of this might be the design of trials aimed at improving the welfare of farmed animals (e.g., dairy cows) by testing different farming conditions (e.g., “high welfare” vs. “low welfare” settings) or different health and welfare monitoring devices. In this case, the research would be relevant for the species, besides being potentially beneficial to a large number of other dairy cows, including some of the individuals involved in the trials, should the techniques under test be found to be successful.
- *Impartiality:* This means that animal-centered research should afford protection to all partakers as a consequence of their role in the research and not of their characteristics (e.g., species, sentience) or societal practices (e.g., food vs. companion animals). In other words, researchers should try to avoid any bias due to taxonomic classifications, societal considerations and human-centered preferences (e.g., dogs vs. pigs in Western cultures); instead, they should endeavor to acknowledge and respect the individual characteristics of those who partake in research procedures regardless of their species. The authors argue that treating partakers as individuals equally deserving of consideration and care according to their welfare needs (see also the principle below) would yield benefits both from an ethical and from a scientific point of view, avoiding any underestimation of the participants’ abilities and sensibilities; and, consequently, any possible methodological bias which could threaten the validity of research findings. For example, research that aims to investigate and compare the cognitive capacities of different animal species needs to treat all animals involved equitably, and employ species-specific methods and tools which respect their characteristics and, thus, allow them to express their potential.
- *Welfare:* According to this principle, when carrying out any harm-benefit analysis and considering the potential impact of a procedure, the focus should be on the individual animals’ (or their species’) best interests, and these interests should prevail over the interests of science and society. In other words, to support animal-centered research, partakers’ welfare should be prioritized at all times. In this regard, researchers should consider the animals’ biological integrity (i.e., their physical and psychological health) and autonomy (i.e., their ability to express and pursue their wants) without disrupting their daily life patterns and routines, instead granting the greatest possible degree of freedom of expression and control over both the research environment and the research process, and respecting the animals’ needs and wants. An example of practice clearly in contrast with this principle would be, for example, housing social species (such as rats or mice) in individual cages or in barren environments to prevent them from interacting, or withdrawing food for a prolonged period of time before blood collection.
- *Consent:* This principle requires that individuals’ informed consent always be garnered, comprising two complementary and both necessary forms: *mediated* consent from legal guardians and welfare experts (or ethical review bodies) in the animals’ best interests, and *contingent* consent from the animals themselves, as expressed by their willingness to engage and chosen modalities of engagement. This principle might indeed be the one generating more discussion. Akin to ethical approaches to research involving human subjects, partakers in animal research should be afforded sufficient control to allow them to make relevant choices, including the choice not to engage or to decide the pace and modality of their engagement with a research process, at any time. Possible means to achieve this might be: allowing as much time as possible for the animals to assess the situation (e.g., in an unfamiliar environment); planning for alternative forms of engagement and allowing animals to make choices (e.g., different interactions with experimental equipment or different reward mechanisms); allowing animals to effectively withdraw or withhold engagement (e.g., by providing escape routes, hiding places or comfortable rest areas, as appropriate). Examples of procedures consistent with this principle might be provided by research that involves dog training for sensitive tasks (e.g., dogs employed in the detection of human diseases); for the training to be effective, dogs need to be allowed to set the pace of work and sessions are immediately interrupted when they stop engaging in the proposed activity or show signs of stress, boredom or tiredness.

Evidently, there is interrelation and, thus, a degree of overlap among the concepts of relevance, impartiality, welfare and consent (e.g., as impartiality and consent increase, animal welfare is likely to increase as well; as relevance increases, consent and welfare are also likely to increase). However, each concept emphasizes a distinct ethical concern (as discussed in section 2.2), thus accounting for the potential impacts of the research on animals from different angles, which are not usually considered by commonly used animal welfare assessment schemes. For example, the concept of impartiality accounts for the possibility that some of the partakers’ needs might not have been scientifically demonstrated yet but might nevertheless still warrant consideration (e.g., might insect larvae be sentient and able to experience pain?). For another example, the concept of consent emphasizes partakers’ agency and the importance of considering whether and how they might be allowed to withdraw from the experimental procedures. Also, although there might be cases in which an animal’s ability to dissent would be detrimental to their welfare (e.g., if they were allowed to refuse medication that would alleviate their symptoms, thus negatively affecting their wellbeing), the psychological impact of overriding the animal’s dissent would be part of the “welfare equation.” In other words, the ACRf principles are at the same time interrelated and complementary, bringing into focus distinct ethical concerns and their interactions. A summary of the principles and their main characteristics is shown in Table 1.

**Table 1:** Principles of animal-centered research and scoring system, based on a scale ranging from 1 to 5, where 1 is “very low” (VL), 2 is “low” (L), 3 is “moderate” (M), 4 is “high” (H) and 5 is “very high”(VH)—adapted from Mancini and Nannoni (4).

Principles		Relevance	Impartiality	Welfare	Consent
Definition		Procedure is directly relevant to and beneficial for all partakers	Partakers receive the highest consideration regardless of their capacities	Procedure is fully compatible with or enhances partakers’ welfare	Partakers are enabled to choose whether and how to engage with procedure
Scoring scale	5 (VH)	Procedure is directly relevant and highly beneficial for partakers	Individuals receive highest consideration regardless of their capacities	Procedure enhances partakers’ welfare	Partakers are enabled to choose whether and how to engage with procedure
	4 (H)	Procedure is relevant for partakers but benefits may not be direct or immediate	Individuals receive high consideration but not as much as others with more capacities would	Procedure does not impact negatively on partakers’ welfare	Partakers are mostly able to choose whether and how to engage with procedure
	3 (M)	Procedure has some relevance for partakers but benefits are only indirect and only in future	Individuals receive some consideration but notably less than more capable ones would	Procedure has minor negative impact on partakers’ welfare	Partakers have limited ability to choose whether and how to engage with procedure
	2 (L)	Procedure has little relevance for partakers and benefits are only indirect and only in future	Individuals receive significantly less consideration than more capable ones would	Procedure has significant negative impact on partakers’ welfare	Partakers are mostly not allowed to dissent or withdraw from procedure
	1 (VL)	Procedure has no relevance whatsoever and no benefits for partakers even indirectly or in future	Individuals receive very little or no consideration compared to more capable ones	Procedure has severe negative impact on partakers’ welfare	Partakers are not allowed to dissent or withdraw from procedure in any way
Additional notes		Procedures that are directly relevant to the animals involved allow to achieve more relevant, useful and directly applicable results. Relevance should primarily pertain to the individuals involved but, less directly, could also pertain to the species or category, whose interests are represented by the animals involved.	Researchers should be mindful of and endeavor to avoid social, cultural and legal bias affecting different human-animal relations, and all research participants should comparatively receive the same consideration as the most protected species involved in research (i.e., humans).	This could include the design of milder procedures that guarantee higher welfare; provisions for re-homing participants at the end of trials; research set-ups that allow participants to exert control over the environment. Welfare should be assessed by also using positive welfare indicators.	Although this principle raises difficult issues regarding the possibility of enabling animals to provide and humans to interpret consent, it is a critical animal-centered consideration. Although the principle might not be applicable in some cases, the ethical conundrum should be acknowledged anyway. Ideally consent should be a continuous process to be negotiated with participants, as opposed to a one-off approval given by ethical review bodies.

Together with the four ACRf principles, Mancini and Nannoni (4) propose a system for scoring procedures against each of the principles on a 1-to-5 (from very low to very high) scale, to help assess the extent to which the design of a research procedure might be consistent with an animal-centered perspective. The authors illustrate the possible application of their scoring system on different examples of published research, as a starting point from which to further articulate the framework’s applicability. The work presented here builds on and advances the authors’ previous work, aiming to demonstrate how the ACRf might be operationalized and integrated within a comprehensive ethical review process. To this end, the following sections describe how the ACRf scoring system can be used as a method to assess the design of a research procedure and as a tool to facilitate an iterative process of refinement within a new ethical review flow. This is subsequently demonstrated through a theoretical case study of a research trial on pigs’ response to stress.

## 3 Methods

### 3.1 Scoring of experimental protocols according to the ACRf

The ACRf scoring system is shown in Table 2, where an example experimental procedure for each score is provided. However, the table provides only an overall reference scheme based on a very general, broad definition of each intended animal use. For this reason, the scoring system presented here should be used by the relevant stakeholders exclusively to familiarize themselves with the framework. A full framework application will instead necessarily need to be based on a considerably more detailed description of the experimental procedures to be carried out and on the refinement strategies to be adopted. That said, the following provides indicative criteria for using the scoring system:

**Table 2:** Scoring system method and classification examples according to the animal-centered research framework.

Score	Relevance	Impartiality	Welfare	Consent
**5**	Procedure is relevant for partakers	Individuals receive highest consideration regardless of their capacities	Procedure enhances partakers’ welfare	Partakers are enabled to choose whether and how to engage with procedure
***e.g., Using a drug to reduce participants’ existing disease symptoms***	*Partakers already suffer from a spontaneously occurring disease and the trial is set to benefit them individually and directly*	*Partakers receive a high standard of care, they are allowed to freely express and meet their physical, behavioral and emotional needs at any time*	*Experimental setting improves the living conditions of partakers compared to standard living and housing conditions* (e.g.*, providing housing with no height differences, stairs or slopes for animals with impaired mobility*)	*Partakers are allowed to choose between different medicated or unmedicated foods and their spontaneous response is recorded*
**4**	Procedure is relevant for partakers but benefits may not be direct or immediate	Individuals receive high consideration but not as much as others with more capacities would	Procedure does not impact negatively on partakers’ welfare	Partakers are mostly able to choose whether and how to engage with procedure
***e.g., Testing several painkiller drugs to identify the most effective one***	*Partakers suffering from a painful condition may or may not benefit from the trial depending on which painkiller they receive but findings are set to benefit the species and all partakers in future*	*Partakers receive a good standard of care, some aspects are not properly considered* (e.g.*, poor enrichment*) *because they are thought to be irrelevant for the species or for the duration of the trial*	*Animals receive a standard level of housing and care; their welfare is not affected by experimental procedures*	*Some procedures/sessions are scheduled to assess painkillers’ effect (including animal manipulation and samples collection) but partakers can decide when to stop them*
**3**	Procedure has some relevance for partakers but benefits are only indirect and only in future	Individuals receive some consideration but notably less than more capable ones would	Procedure has minor negative impact on partakers’ welfare	Partakers have limited ability to choose whether and how to engage with procedure
***e.g., Testing promising experimental drugs in vivo for the first time (effects on the species are not entirely known)***	*Partakers may be harmed during the trial, and they and their species may never benefit from the research findings*	*The standard of care is poor, some aspects are lacking* (e.g.*, enrichment, social contact*) *because they are thought to be irrelevant for the species*	*Partakers experience minor discomfort of short duration due to the procedures*	*Partakers are trained to sit or to stand still for a blood drawing because they will receive a treat afterwards*
**2**	Procedure has little relevance for partakers and benefits are only indirect and only in future	Individuals receive significantly less consideration than more capable ones would	Procedure has significant negative impact on partakers’ welfare	Partakers are mostly not allowed to dissent or withdraw from procedure
***e.g., Testing a drug intended for another species but which may be used in the future also in the partaking species***	*Partakers are unlikely to benefit from the research findings but are likely to suffer harms during the trial, even though their species might benefit in future*	*The standard of care is low but animals are checked for signs of poor welfare*	*Partakers are likely to experience moderate discomfort or pain during the procedures*	*The procedure* (e.g.*, blood drawing*) *is carried out unless the animal shows signs of distress*
**1**	Procedure has no relevance whatsoever and no benefits for partakers even indirectly or in future	Individuals receive very little or no consideration compared to more capable ones	Procedure has severe negative impact on partakers’ welfare	Partakers are not allowed to dissent or withdraw from procedure in any way
***e.g., Testing the toxicity of a drug for human use***	*Partakers are knowingly harmed during the trial and will never benefit from the research findings*	*A minimal standard of care is adopted because animals are deemed incapable of experiencing discomfort or pain*	*Partakers are likely to experience severe and prolonged discomfort or pain during the procedures*	*Partakers are restrained and the procedure is carried out as programmed*

For ***Relevance***, procedure scores vary from a minimum level (1) where the procedure does not have relevance or bring benefits to the partakers, even in the future or indirectly (e.g., regulatory studies testing a drug intended for human use on rodents) to a maximum level (5) for procedures that are directly relevant and beneficial to the individuals taking part in the research (e.g., testing a drug to reduce the symptoms of a spontaneously occurring disease, which could improve partakers’ conditions). Intermediate scores can be used for research expected to be relevant and/or provide benefits only to some of the participants (e.g., only participants receiving a specific drug) of for research whose benefits to the individuals involved might be indirect or only come to fruition in the long term (e.g., testing little known drugs or drugs primarily intended for other species).

For ***Impartiality***, the highest score would be given to procedures that give greatest consideration and provide the highest possible standard of care to participants regardless of their species, affording not only freedom from discomfort and pain but also freedom to express individual physical, behavioral and emotional needs. Progressively lower scores would be given to procedures that give lower consideration and provide less comprehensive and less than optimal standards of care if the species in question is deemed to have lower moral standing. The minimum score would be given to those procedures for which a very low standard of care is adopted because animals are deemed incapable of experiencing discomfort or pain, or are not the subject of societal concern, and are thus attributed lower moral standing (this could be the example of insects, larvae or some invertebrate species used for research).

For ***Welfare***, the score would increase the more partakers’ welfare is taken into consideration and the experimental trial is adapted to their individual and species-specific needs, up to a maximum score (5) for cases in which the welfare of the partakers is even improved by their participation in research. This could be, for example, the case when, by being involved in a study, participants acquire new capabilities that enabled them to enrich their cognitive experience; or when, during a study, animals are kept under conditions that are ameliorative compared to the husbandry conditions habitually experienced by animals for their same category (e.g., food vs. companion pigs) and species (e.g., a laboratory pig taking part in behavioral research compared to a commercially housed pig intended for food production), provided that the favorable conditions persist once the research has concluded. The lowest score on the scale (1) would be obtained by those studies where partakers’ welfare is severely compromised by their involvement.

Similarly, for ***Consent*** the minimum score would be attributed to procedures to which partakers are not enabled to express (or withdraw) their consent (e.g., being restrained and subjected to a drug administration or a blood withdrawal); whereas the highest score will be given to those research procedures where partakers can freely choose whether, how and for how long to participate (e.g., dog training sessions that enable partakers to engage as they prefer and that are immediately interrupted if they show signs of disengagement).

### 3.2 The ACRf as a refinement tool

The scoring method described above could be used to foster increasing attention toward wider ethical concerns to help researchers and delegated authorities assess the extent to which research procedures align with these principles and determine when being involved in research is in an animal’s best interests, when a procedure could be adjusted to increase its ethical standard or when the use of non-animal methods is more urgently advisable. The exercise of applying the framework to a prospective research procedure could stimulate reflection on possible alternative research designs aiming to refine not only the procedures to be carried out using animals, but also the ethical review process as a whole.

Generally speaking, refinements can be classified as science-driven (they facilitate getting high-quality results) or welfare-driven (they are put in place to minimize or alleviate animal suffering) (32). As mentioned earlier, refinements can cover a wide range of aspects, for example: using improved methodology in invasive techniques, adopting less invasive experimental protocols, administering analgesic and/or anesthetic drugs, reducing the number of samples to be taken from the animals, guaranteeing adequate housing and husbandry to satisfy the animals’ physiological, psychological and ethological needs, ensuring proper expertise and training of staff dedicated to experimental or husbandry activities, etc. However, to the best of our knowledge, no standardized approach is available to organically assist ethical review bodies in assessing whether the range of possible adaptations that could be made to research protocols under the umbrella of “refinement” is, or is not, adequate. This is in contrast with what happens for the other two Rs, where a more structured approach is generally applied. The principle of “replacement” is clearly defined, and its implementation can be assessed by the fact that, whenever possible, *in vivo* techniques previously used, or potentially usable, for a given procedure are discounted in favor of *in vitro* or *in silico* methods; or that species previously used, or potentially usable, for a given procedure are substituted with species deemed to be less likely to suffer related harms due to their biological characteristics and related capacities. Similarly, the principle of “reduction” is clearly defined and its implementation can be verified based on whether the number of animals used is the minimum required to obtain statistically sound experimental results.

In contrast, assessing the implementation of “refinement” is less straightforward because, as mentioned, refinement strategies often imply the adoption of multiple interventions covering several aspects [e.g., (33)]. This variety can be hardly assessed by considering each intervention separately and requires instead a more comprehensive/transversal approach. More fundamentally, as discussed, available refinement guidelines only focus on welfare harms [e.g., (7, 8)] with no consideration for broader *ethical harms*. In this regard, the ACRf could be used as an integrative tool within the current ethical review practices, in particular to systematically assess the refinement of research protocols pertaining to its four principles.

Ideally, the assessment of refinements would be outcome-based (i.e., based on the expected effects of the refined research protocol on the subjects involved) rather than design-based (i.e., based on the list of proposed modifications in the design of the research), in order to evaluate whether the efforts made to refine an experimental procedure can be deemed sufficient or whether further work is needed to achieve a desirable refinement outcome (34). However, the ACRf could serve as a tool to refine research designs and assess any refinements before these are implemented and before the resulting outcomes are evaluated.

**Figure 1:**
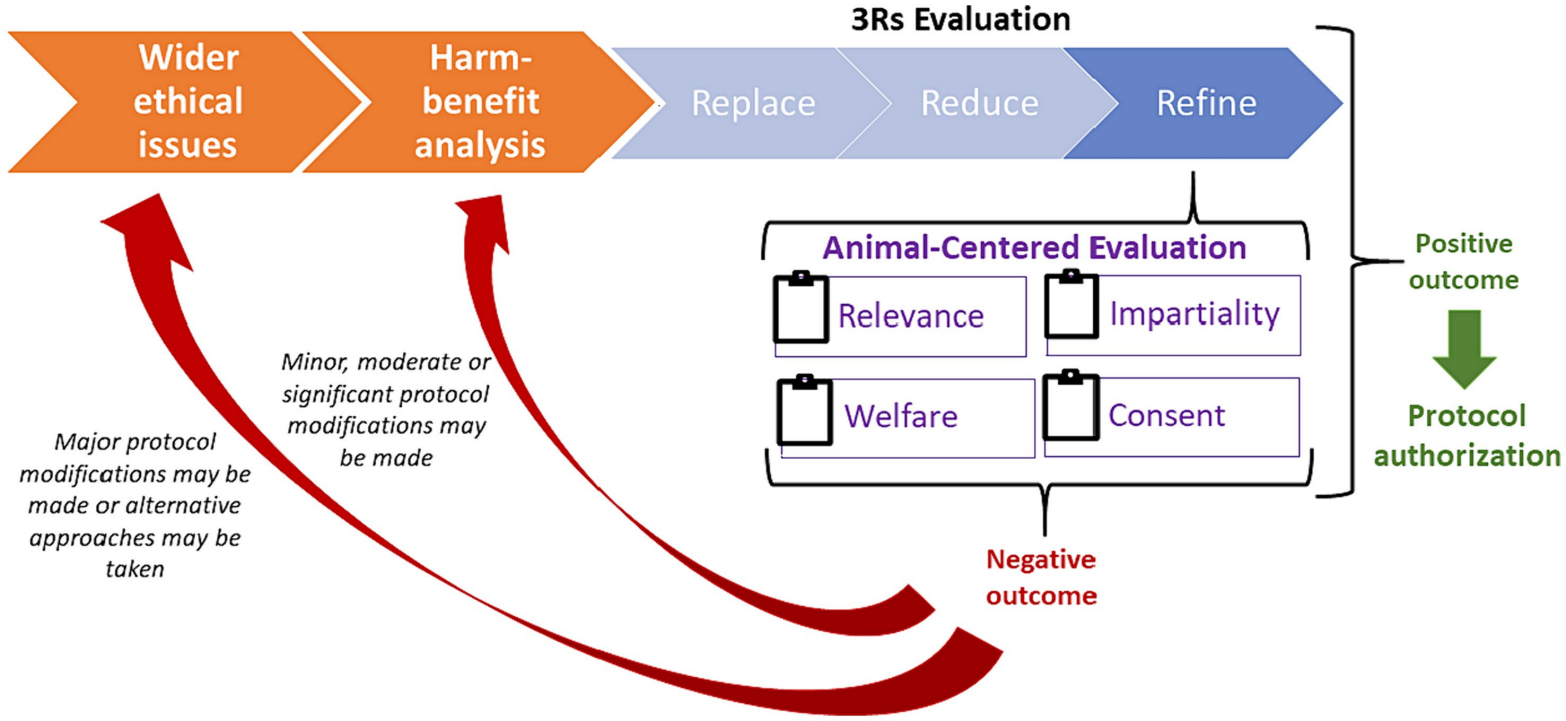
The iterative ethical review process integrating the ACRf principles to help determine whether and how proposed research should be refined, significantly modified or abandoned in favor of alternative approaches.

Thus, we propose a new flow for the ethical review process where, as shown in Figure 1, when carrying out the assessment of a research protocol based on the 3Rs framework, the ACRf could be used as a complementary method to evaluate in a more systematic way whether a research protocol needs to be refined or whether it should be altogether reconsidered. For each of its constituting principles (Relevance, Impartiality, Welfare and Consent), the ACRf would require an evaluation of the research protocol in question on a 1-to-5 scale (where 1 = “the protocol raises serious ethical concerns” and 5 = “ethical concerns are minimal or have been properly addressed”). Based on this evaluation, a threshold could be established below which a protocol should be reconsidered. For the sake of argument, we propose that, if the research protocol scores at the very least 70% against the ACRf (= at least 14 points out of 20), the research protocol in question could be regarded as sufficiently refined to allow the project to undergo the rest of the ethical review. On the contrary, should the protocol score below the minimum threshold, its reconsideration would be necessary, primarily in terms of replacement and reduction if the ethical concerns raised are serious, and secondarily in terms of additional refinements. This approach would emphasize and facilitate the iterativity that, to a limited extent, already characterizes the ethical review process, by making subsequent adaptations and refinements a central, traceable part of the process to be carried out by means of a back-and-forth discussion between the researchers proposing the protocol and the ethical review body responsible for assessing it.

As shown in Figure 1, wider ethical considerations (regarding the appropriateness of using animals in research) and harm-benefit analysis (whether expected harms are warranted in view of expected benefits) are pre-requisites to the proposed application of the ACRf and are to be preliminarily assessed in the research design phase. However, opportunities for refinements identified during the application of the ACRf may result in significant changes in the experimental design. In turn, this might lead to a re-assessment of the harm-benefit balance (e.g., if refinements had eliminated or reduced previously expected harms) and, consequently, of wider ethical consideration (e.g., if animals were granted impartial treatment and the opportunity to consent to research beneficial for them and compatible with their welfare), as part of the iterative process depicted in Figure 1.

As discussed, iteration is an essential characteristic of the design process when developing animal-centered technology within ACI, ensuring that the requirements of animal stakeholders are gradually uncovered and adequately met. In the process, the application of design principles is instrumental in ensuring that prototypes are sufficiently refined before animal research participants are exposed to them. Similarly, iteration should be an essential characteristic of the research design process for any research involving animals, to ensure that this is as animal-centered as possible. In this regard, the ACRf provides principles that could iteratively inform the refinement of research designs as far as possible before piloting them with animals (35).

As previously discussed by Mancini and Nannoni (4), in some research scenarios it might be challenging or impossible to refine research procedures through the application of the ACRf, for example, in the case of regulatory tests that are known to cause severe harm to the animals being used but are nevertheless required by law (e.g., toxicological studies). However, in those difficult cases, scores obtained using the ACRf could at least be used as a benchmark, stimulating additional ethical reflection and promoting a continuous improvement in research design, with the ultimate aim to refine these by including also the perspective of the animals as primary stakeholders, rather than scientific instruments.

To illustrate how the proposed framework could be operationalized, the following sections describe a theoretical case-study involving the use of pigs in animal welfare research. The case-study is firstly discussed in relation to the three facets of the ethical review process (harm-benefit analysis, wider ethical issues and 3Rs). Then the case study is discussed through the lens of the ACRf, providing an example of how the integration of the framework could support a holistic and iterative ethical review process.

## 4 A case-study application: description and results

This section presents the case-study, a related ACRf-based assessment and resulting modifications to the research protocol. The main aspects of the case-study analysis are summarized in Table 3.

**Table 3:** Analysis of a case study on a research protocol on pig welfare: application of the 3Rs principles and ACRf scoring system.

**Assessment with the 3Rs Framework**	**Replace:**		**Reduce:**		**Refine:**		Unlikely if the aim is to gain knowledge on pig stress response.		The number of animals has been reduced to a minimum without compromising the statistical power.		Reasonable measures will be taken (personnel training, look for signs of distress, anesthetic cream). Do all the procedures need to be made (especially the biopsy)?	
		**Relevance**	**Impartiality**	**Welfare**	**Consent**
**Scoring using the ACR framework**	Moderate: **Score 3**	Moderate: **Score 3**	From moderate to high depending on the group: **Score 3 to 4**	Very low: **Score 1**
**ACRf total score—1st assessment**	**10–11/20—The protocol needs improvement**			
**Proposed protocol modifications after the ACRf assessment**	Carrying out periodical and individual health checks during the training sessions (see the “consent” column). Improving adherence to “real life” condition (use of transport to slaughter as a stress challenge).	Acknowledging the ethical issue given the protocol constraints.	Using only one of the stressful procedures (i.e., only transportation and not mixing). Affording care provisions as needed following health checks.	Training animals (with positive reinforcement) to voluntarily undergo blood sampling and health checks.
**New score after the modifications**	High: **score 4**	Moderate: **score 3**	High: **score 4**	Moderate: **score 3**
**ACRf total score—2nd assessment**	**14/20—The protocol is satisfactory**			

### 4.1 Case-study description

A group of researchers studying pigs’ response to stress are preparing a research protocol. They would like to assess whether high-welfare farming systems (late weaning, provision of additional space and rooting materials) can reduce the pigs’ stress response. The study is important for multiple reasons: promoting good welfare conditions on farms, possibly reducing stress response during transportation (which is one of the most stressful phases in farm animals’ lives) and limiting the negative effects of transportation—and the associated stress response—on meat quality. To this aim, their experimental protocol involves raising two experimental groups of pigs. The first group is housed in standard Commercial Conditions for farmed pigs (CC) and the other one is raised in a High Welfare system (HW), which is specifically created for this trial as it does not correspond to standard rearing practices adopted in commercial farms. The animals remain in the respective farming system for 3 months during which they are periodically weighed, and are then subjected to a stressful challenge in order to evaluate their physiological response. The challenge (exactly the same for both experimental groups) consists in mixing pigs with unfamiliar conspecifics coming from the same housing system, loading them on a truck and transporting them for approximately 2 h, using standard commercial practices and equipment. To avoid confounding factors, the personnel handling and transporting the pigs is the same for the two experimental groups. The animals are then unloaded and various physiological samples are taken. A blood sample is collected by jugular vein puncture for the analysis of blood stress parameters, a hair sample is collected by shaving the rump region to assess chronic stress levels using cortisol levels in bristles, and a skin biopsy is carried out to assess the possible presence of alterations in the peripheral immune response due to chronic stress. Lastly, a visual count of skin lesions is conducted and animals are monitored through video recording for 2 h to carry out behavioral observations.

The protocol falls under the Directive 2010/63/EU on the protection of animals used for scientific purposes (36). The research protocol undergoes the ethical review process prescribed by the Directive and the researchers submit the experimental protocol to the local AWERB for its perusal and authorization, as required by the Directive. Below we report a summary of the most relevant comments the researchers could provide in the ethical review application.

***Harm-benefit analysis.*** The main benefit of this project would be to gain new knowledge on the relationship between animal welfare conditions and stress response. In the long term, this knowledge could potentially lead to improved animal welfare conditions on farms, should the results obtained prove a less intense stress response in the animals raised under HW conditions. The experimental farming conditions cause no significant harm to the animals, since they are raised either under standard or HW conditions. The more likely harm inflicted upon the animals would be the stress challenge (transportation under commercial conditions) and the blood and biopsy samples collection, whereas all the other manipulations (weighing, visual lesions assessment, hair sampling, video recording) would be unlikely to cause harm.

***Wider ethical issues.*** The increased knowledge obtained through the trial would benefit the scientific community and help advance understanding of an important issue in animal welfare science (i.e., the effects of previous welfare on stress response). This would be all the more important considering that, according to the research team, there is a lack of previous studies on these issues. Importantly, the involvement of animals in this kind of research is essential, since the very object of the investigation is the animals’ responses to different environmental and management conditions, and how these impact their welfare.

***Three Rs implementation.*** Replacement cannot take place in this kind of studies since the aim is precisely to assess a species’ response to a predetermined set of events (farming conditions, transportation, etc.). However, the application of the Reduction principle has led researchers to lower the number of animals to be used, thanks to pre-existing research on the assessment of physiological parameters in stressed pigs. In particular, researchers decided that only a subsample (50%) of the experimental subjects will undergo blood drawing and skin biopsy. Lastly, with respect to Refinement, researchers have planned carefully their sample collection procedures, foreseeing that they will be carried out by trained and experienced personnel, and they will be stopped immediately if any pig shows signs of distress. In addition, the biopsy will be carried out after the application of an anesthetic cream.

Based on the application submitted by the research group, the AWERB deems the harm-benefit analysis to be adequate, the wider ethical considerations to be a bit succinct and the application of the three Rs to be fit for purpose. However, the Review Body expresses a concern related to the biopsies, deeming their need to be not adequately substantiated by the literature provided by the researchers. In a rebuttal letter, the researchers agree to remove the procedure since it would provide complementary information but would not be essential for the purposes of the trial. They take this decision also considering the invasiveness of the procedure and the limited amount of literature on its application to stress challenge studies. The revised ethical protocol is approved by the AWERB.

### 4.2 Assessment according to the animal centered research framework

The research protocol scores a 3 in terms of relevance: while half of the partakers benefit from an improvement in their living conditions, part of the animals undergoes blood sampling and all of them are subjected to loading, transportation and unloading which does not bring any benefit. In addition, the research does not directly aim to be beneficial for the species but to increase the knowledge on physiological responses. The score for impartiality is also 3: while on farm, animals receive either an adequate standard of care for farmed animals or an improved one, but they are unlikely to be able to fully express themselves at all times. The overall score for welfare is 3 or 4 depending on the experimental group (C or HW, respectively), based on the balance between farming conditions and the need to undergo transportation and sampling. Lastly, the protocol scores very low (1) on consent because animals are never allowed to choose whether to take part or not in the research protocol and would probably dissent to some procedures (blood sampling, transportation). The overall score under the ACRf is of **10–11 points out of 20**.

Due to the low score obtained, according to the ACRf researchers should be encouraged to improve the ethical value of the research protocol by additional means, for example:

- As concerns **consent**, the most challenging principle, researchers could implement strategies to provide pigs with a certain degree of control and capability to express consent to the blood sampling procedure: a training and reinforcement schedule could improve animals’ willingness to undergo the sampling procedure even though a certain degree of restraint would still be necessary.
- With respect to **welfare**, it should be discussed whether the stressful conditions (mixing and transportation) need to both be carried out or whether only one of them would be sufficient. As an alternative, a stressful event that would occur anyway could be standardized and used (e.g., transportation to slaughter, if pigs are raised on conventional farms – however, it is open to discussion, whether the training procedure carried out on farm would be able to reduce the animal’s stress during blood sampling at the slaughter plant).
- The **relevance** of the protocol, in this case, is intertwined with the welfare level of the animals. On the one hand, animals involved in the procedure are unlikely to directly benefit from taking part in the research protocol. However, standardizing and using as a stressful challenge an event that would happen anyway in the life of the animals as described above would improve the relevance of the protocol for the species since it would make its setting more similar to “real-life” conditions, therefore making its results (better understanding of the stress response and of how they are affected by different farming systems) potentially more applicable for the population of farmed pigs. In addition, relevance to the animals taking part in the experiment could improve if the training sessions to habituate animals to blood sampling (possibly associated with positive reinforcement) were used also as an opportunity to check the animals’ health and welfare state and identify possible problems and any appropriate interventions (e.g., medical treatment, exclusion from the study). This approach would therefore improve compliance with three of the four ethical principles.
- In terms of **impartiality**, this example does not describe in detail the standard of care of the animals. However, given the specific experimental parameters (housing under “standard” commercial conditions vs. housing under “high-welfare” conditions), we may assume that their treatment, while being respectful of legal requirements for the protection of pigs (i.e., adequate in terms of welfare), would be unlikely to afford them equal freedom of expression and equal provisions for the physical, behavioral and emotional needs of both groups. In other words, by experimental requirement, these animals are not expected to receive equal consideration, let alone receiving the same consideration that research subjects afforded greater protection (e.g., primates, humans) would receive. Thus, in this case study, not much could be done to refine the experimental procedure and increase compliance with the impartiality principle, apart from acknowledging that this is one of the most challenging aspects for this protocol and encouraging researchers to explore the possibility of taking and entirely different approach to address similar research questions in future.

### 4.3 Modified protocol after the ACRf assessment

Overall, as mentioned above, we have proposed that, when using the ACRf, researchers should aim for a score of at least 14. In this case the Researchers, based on the analysis presented above, decide to modify their research protocol in four ways:

1. using only transportation as a stressful factor and avoid mixing;
2. instead of transporting animals only for the purposes of the trial, transporting animals one time only at the end of their production cycle and carrying out the sampling and the behavioral observations at the slaughter site;
3. confirming the exclusion of the skin biopsy, as suggested by the AWERB, since the procedure would not be relevant for the animals involved in the research (and the main aim of the experiment would likely be reached also without this additional procedure);
4. training the animals to collaborate during blood sampling using positive reinforcements, in order to reduce stress during blood collection and providing health checks and care as needed at the same time.

These four modifications substantially increase the scoring of the protocol against the *welfare* principle (fewer procedures, no mixing). The protocol can now be scored 4 against welfare. *Relevance* is also slightly increased due to the overall conditions (transport to slaughter only), which is more consistent with the common conditions commercial pigs would experience during their life. Compliance with relevance is also increased by the provision of health checks and care during blood sampling. However, even provided that neither group of pigs suffers any additional harms due to the experimental procedures, only one half of the animals (the HW group) will experience direct benefits from the procedure, so the score against this principle cannot go above 4. Scoring against *consent* is improved by training the animals for blood drawing, thus reaching a score of 3. The score for impartiality remains 3. The ACRf assessment of the protocol now yields a final score of 14.

While barely reaching the threshold value of 14, the application of the ACRf has informed a significant improvement of the research protocol’s score, by encouraging the researchers to reconsider their experimental design based not only on welfare requirements, but also on wider ethical aspects, therefore resulting in a more favorable *harm-benefit analysis*: stressful procedures would be reduced and the overall study design would limit the number of procedures to be carried out and mimick in most cases common farming and transportation conditions, making the results of the trial potentially more applicable to real-life conditions. While considering the *wider ethical issues,* the protocol revised according to the four ACRf principles would provide a more considerate use of animals, addressing (or at least acknowledging) key ethical issues arising from the specific research protocol, also taking into account otherwise poorly considered aspects such as relevance and consent.

## 5 Discussion

The above case-study advances the previous work of Mancini and Nannoni (4) by contextualizing the application of the ACRf within the broader picture of the ethical review process and by illustrating the operationalization of the ACRf through the theoretical application of its four principles to the ethical review of a study of pigs’ response to stress.

As is often the case in interaction design, the case-study we described here presented several constraints (i.e., fixed conditions that could not be changed). For example, since pigs were intended for food production, there would be no alternative to slaughtering them at the end of their commercial life. Nevertheless, this case study highlights how, even within a commercial setting, it would be possible to reconsider research protocols and make the experimental trials, not only less damaging for the animals themselves (consistent with the 3Rs approach and the harm-benefit analysis), but also more sustainable from an ethical standpoint. In our case study, this was achieved by envisaging that the animals could be allowed to express their agency by participating in positive reinforcement training, which would give them the opportunity to consent to blood sampling procedures, albeit within significant experimental constraints. Greater ethical sustainability was also achieved by associating health checks and care measures as appropriate to the blood sampling procedures, thus increasing the relevance of said procedures for the animals involved, in addition to providing for better welfare.

The case study also illustrates how the inability of a research protocol to achieve maximum score against one or more principles may intrinsically depend on necessary experimental conditions. For instance, the criterion of impartiality could not be met, not only because overall the animals’ treatment was not equitable (even considering species-specific needs) to the treatment that human research participants would receive (e.g., by being allowed to withdraw); more fundamentally, the study required that the two groups of pigs be kept under different housing conditions, which would have been more favorable for one of the groups, resulting in inherent partiality within the study. The issue of “partiality within studies,” which is likely to arise with any studies that require comparison between an experimental and a control condition, could be addressed by adopting a definition of *relative impartiality*: impartiality could be achieved, not necessarily only when different groups of participants in a study are treated equally, but also when they are treated justly, that is, when their needs are met to a minimum threshold of adequate fulfillment above which inequalities in their treatment are not unjust (37, 38). Such a notion could provide greater flexibility in the application of the principle, while maintaining its value and without affecting the value and application of the other principles.

For the sake of argument, in our case study we established that a total score of at least 14/20 against the four principles of the ACRf should be achieved by the envisaged procedure before it could be deemed ethically permissible. Of course, we acknowledge that this threshold is arbitrary and suggest that it could be set depending on the framework’s application context. For example, different AWERBs might set different thresholds for different kinds of research, depending on how important the proposed research is deemed. This would not equate to lowering the standard of animal-centeredness envisaged by the framework: a procedure scoring 20/20 would still be animal-centered whereas a procedure scoring 12/20 would only be half-way there. However, setting a threshold for different kinds of procedure, could give ethical review bodies a criterion to authorize a procedure or prohibit it and ask through another refinement cycle. This would acknowledge that, for some procedures, achieving a 20/20 score would simply be impossible but would also be a way of ensuring that at least the set score for that type of procedure is achieved before the procedure can be authorized. In this regard, the ACRf provides a means to assess the “distance” of a research protocol from animal-centeredness, thus highlighting any limitations against the four principles, while affording flexibility to those who would need to apply the framework to assess research designs consistently and transparently.

As illustrated by our case study, we propose that the issues arising from the application of the ACRf during the ethical review of a research procedure could feed into a systematic harm-benefit analysis as well as encourage consideration of wider ethical aspects concerning the involvement of animals in research. In other words, we suggest that the integration of the ACRf into the ethical review process has the potential to provide a holistic approach to the ethical assessment of research designs. Its basic constituents (the four principles) and scoring system provide a simple and easy-to-apply method to assess specific aspects of a research design, at the same time stimulating reflection on wider ethical aspects. Its use within an *iterative* ethical review process of progressive refinement would mean that the ethical assessment of a research design could take the form of ongoing, dialogic engagement between researchers and ethical review bodies, rather than a one-shot attempt to obtain ethical approval before the beginning of research trials. While we realize that not all kind of research might be comprehensively assessed using the ACRf, we propose that future work in collaboration with different ethical review bodies could investigate the challenges that might arise from the application of the ACRf to the ethical review process of a wide range of research proposals.

An obvious limitation of the present work is that the operationalization of the ACRf was based on a theoretical, albeit realistic, case-study, which means that its application did not necessarily account for complexities and difficulties that researchers and review bodies might have to deal with in real cases. However, here our aim was to show how the ACRf might fit within, and be used as a supporting tool for the ethical review process that all proposed animal research needs to undergo, and how its application might result in changes to the design of research procedures as well as foster broader considerations. The next step in the development of the proposed approach will be to invite researchers and ethical review bodies to apply the ACRf to real projects, respectively when they prepare research designs and when they assess research proposals, to do which they will benefit from being able to refer to a case study that illustrates the ACRf’s integration within the broader ethical review process. In this regard, this paper represents the link, and provides the necessary transition, between our previous work and the work that we expect to do next as a part of a journey toward developing a solid, validated and viable approach which advances an animal-centered perspective to increase the ethical and scientific value of research involving animals.

## 6 Conclusion

In this paper, we have illustrated how the ACRf, with its constituent ethical principles (relevance, impartiality, welfare and consent) and scoring system could be operationalized within the broader context of an iterative ethical review process. As a method, the ACRf is not intended to interfere with researchers’ autonomy and scientific approach, but rather as a mean to support a holistic approach to ethical review by eliciting reflection on how to improve research designs and experimental protocols, thus helping researchers transition toward a more animal-centered research ethics. We propose that integration of the framework within the ethical review process could help researchers and ethical review bodies to discriminate—from an animal-centered perspective—research procedures that would be unethical, those for which animal-centered refinements would be warranted and achievable (as in the described case-study), and those which should definitely be carried out because they would be relevant and beneficial to the animals involved. We acknowledge that some cases (e.g., regulatory studies) present ethical issues that cannot be solved by the application if the ACRf, but the framework could still be applied as a method to improve the ethics—and science—of animal research and, compared to current ethical review processes, to highlight the animals’ perspective and the urgency to transition to alternative methods not involving animals when their involvement does not benefit them.

## Data availability statement

The original contributions presented in the study are included in the article/supplementary material, further inquiries can be directed to the corresponding author.

## Author contributions

EN: Conceptualization, Investigation, Methodology, Validation, Visualization, Writing – original draft, Writing – review & editing, Data curation, Formal analysis, Project administration, Supervision. CM: Conceptualization, Investigation, Methodology, Validation, Visualization, Writing – original draft, Writing – review & editing, Data curation, Formal analysis, Project administration, Supervision.

## Footnotes

^1^The word “partakers” aims to emphasize animals’ subjectivity and agency in the research process, and to signal a shift in perspective away from regarding them as research instruments.

## References

[1] Russell W Stratton M Burch RL. The principles of humane experimental technique. London: Methuen (1959).

[2] Ferdowsian HR Beck N. Ethical and scientific considerations regarding animal testing and research. PLoS One. (2011) 6:e24059. doi: 10.1371/journal.pone.0024059, PMID: 21915280 PMC3168484

[3] Beauchamp TL. Principles of biomedical ethics / tom L. Beauchamp, James F. Childress. Oxford: Oxford University Press (1994).

[4] Mancini C Nannoni E. Relevance, impartiality, welfare and consent: principles of an animal-centered research ethics. Front Anim Sci. (2022) 3:186. doi: 10.3389/fanim.2022.800186

[5] Mancini C. Animal-computer interaction: a manifesto. Interactions. (2011) 18:69–73. doi: 10.1145/1978822.1978836

[6] Richmond J. The 3Rs-past, present and future. Scand J Lab Anim Sci. (2000) 27:84–92. doi: 10.23675/sjlas.v27i2.19

[7] Animal Procedures Committee (APC). ‘Review of Cost-Benefit Assessment in the Use of Animals in Research’. (2003). Available at: https://assets.publishing.service.gov.uk/media/5a7b35a7e5274a319e77dc1d/cost-benefit-assessment.pdf.

[8] Davies Gail F Golledge Huv Hawkins Penny Rowland Anna Smith Jane Wolfensohn Sarah. ‘Review of harm-benefit analysis in the use of animals in research’. Home Office. (2017). Available at: http://hdl.handle.net/10871/31153.

[9] Paci P Mancini C Blaine AP. ‘Designing for Wearability: an animal-Centred framework’. (2019). In *Proceedings of the sixth international conference on animal-computer interaction*, 1–12.

[10] Ruge L. Mancini C. ‘A method for evaluating animal usability (MEAU)’. (2019). In *Proceedings of the sixth international conference on animal-computer interaction*, 1–12. Haifa Israel: ACM.

[11] RSPCA. ‘What Do We Mean by “Ethics”?’. (2023). Available at: https://science.rspca.org.uk/sciencegroup/researchanimals/ethicalreview/whatdowemean.

[12] DeGrazia D Beauchamp TL. Beyond the 3 Rs to a more comprehensive framework of principles for animal research ethics. ILAR J. (2021) 60:308–17. doi: 10.1093/ilar/ilz011, PMID: 31598694 PMC8633449

[13] Laber K Newcomer CE Decelle T Everitt JI Guillen J Brønstad A. Recommendations for addressing harm–benefit analysis and implementation in ethical evaluation – report from the AALAS–FELASA working group on harm–benefit analysis – part 2. Lab Anim. (2016) 50:21–42. doi: 10.1177/0023677216642397, PMID: 27188276 PMC5815834

[14] Brønstad A Newcomer CE Decelle T Everitt JI Guillen J Laber K. Current concepts of harm–benefit analysis of animal experiments – report from the AALAS–FELASA working group on harm–benefit analysis – part 1. Lab Anim. (2016) 50:1–20. doi: 10.1177/0023677216642398, PMID: 27188275 PMC5815836

[15] Dennis M. ‘Animal welfare assessment grid (AWAG)’. (2015). Available at: https://nc3rs.org.uk/crackit/animal-welfare-assessment-grid-awag.

[16] Honess P Wolfensohn S. The extended welfare assessment grid: a matrix for the assessment of welfare and cumulative suffering in experimental animals. Altern Lab Anim. (2010) 38:205–12. doi: 10.1177/026119291003800304, PMID: 20602536

[17] Mellor David J. Reid C. S. W. ‘Concepts of animal well-being and predicting the impact of procedures on experimental animals’. (1994). Available at: https://www.wellbeingintlstudiesrepository.org/exprawel/7/

[18] Mellor DJ. Comprehensive assessment of harms caused by experimental, teaching and testing procedures on live animals. Altern Lab Anim. (2004) 32:453–7. doi: 10.1177/026119290403201s73, PMID: 23581117

[19] FAWC (Farm Animal Welfare Council). FAWC updates the five freedoms. Vet Rec. (1992) 131:357.

[20] Buchanan-Smith HM Badihi I. The psychology of control: effects of control over supplementary light on welfare of marmosets. Appl Anim Behav Sci. (2012) 137:166–74. doi: 10.1016/j.applanim.2011.07.002

[21] Van Weeghel HJE Bos AP Jansen MH Ursinus WW Groot Koerkamp PWG. Good animal welfare by design: an approach to incorporate animal capacities in engineering design. Agric Syst. (2021) 191:103154. doi: 10.1016/j.agsy.2021.103154

[22] Sharp H Rogers Y Preece J. Interaction design: Beyond human-computer interaction. 5th ed. Indianapolis: Wiley (2019).

[23] Norman D Draper SW. User Centered System Design. New Jersey: Lawrence Erlbaum Associates (1986).

[24] Mancini Clara Li Sha O’Connor Grainne Valencia Jose Edwards Duncan McCain Helen. ‘Towards multispecies interaction environments: extending accessibility to canine users’. (2016). In *Proceedings of the Third International Conference on Animal-Computer Interaction*, 1–10.

[25] Webber S. Carter M. Smith W. Vetere F. ‘Co-designing with orangutans: enhancing the Design of Enrichment for animals’. (2020). In *Proceedings of the 2020 ACM Designing Interactive Systems Conference*, 1713–1725.

[26] Martin CF Biro D Matsuzawa T. The arena system: a novel shared touch-panel apparatus for the study of chimpanzee social interaction and cognition. Behav Res Methods. (2014) 46:611–8. doi: 10.3758/s13428-013-0418-y, PMID: 24311060

[27] Webber S Cobb ML Coe J. Welfare through competence: a framework for animal-centric technology design. Front Vet Sci. (2022) 9:885973. doi: 10.3389/fvets.2022.885973, PMID: 35847650 PMC9280685

[28] Mellor DJ Beausoleil NJ Littlewood KE McLean AN McGreevy PD Jones B. The 2020 five domains model: including human–animal interactions in assessments of animal welfare. Animals. (2020) 10:1870. doi: 10.3390/ani10101870, PMID: 33066335 PMC7602120

[29] Coe Jon‘Embedding environmental enrichment into zoo animal facility design’. (2017). In: *Proceedings of the Zoo Design Conference, Wroclaw, Poland*, 5–7.

[30] Makinde Ayoola Islam Muhammad Muhaiminul Scott Stacey D. ‘Opportunities for ACI in PLF: applying animal- and user-Centred design to precision livestock farming’. (2019). In *Proceedings of the Sixth International Conference on Animal-Computer Interaction*, 1–6. Haifa Israel: ACM.

[31] Weeghel HJ Van E Bos APB Spoelstra SF Groot Koerkamp PWG. Involving the animal as a contributor in design to overcome animal welfare related trade-offs: the dust Bath unit as an example. Biosyst Eng. (2016) 145:76–92. doi: 10.1016/j.biosystemseng.2016.02.015

[32] Lloyd MH Foden BW Wolfensohn SE. Refinement: promoting the three Rs in practice. Lab Anim. (2008) 42:284–93. doi: 10.1258/la.2007.007045, PMID: 18625583

[33] Herrmann K. Refinement on the way towards replacement: are we doing what we can? In: H Kathrin J Kimberley, editors. Animal experimentation: Working towards a paradigm change. Leiden, NL: BRILL (2019). 3–64.

[34] Musk GC. Refinements to animal models for biomedical research. Animals. (2020) 10:2425. doi: 10.3390/ani10122425, PMID: 33352864 PMC7766876

[35] Animals in Science Committee. Review of harm-benefit analysis in the use of animals in research. (2017). Available at: https://assets.publishing.service.gov.uk/media/5a81edade5274a2e8ab5695b/Review_of_harm_benefit_analysis_in_use_of_animals_18Jan18.pdf.

[36] EU. Directive 2010/63/EU of the European Parliament and of the council of 22 September 2010 on the protection of animals used for scientific purposes. Off J Eur Union. (2010) L276:33–79.

[37] Nussbaum MC. Frontiers of justice: Disability, nationality, species membership. Cambridge, MA, USA: The Belknap Press of Harvard University Press (2006).

[38] Nussbaum MC. Justice for animals: Our collective responsibility. New York, NY, USA: Simon and Schuster (2022).

